# The organisational climate of NHS Early Intervention Services (EIS) for psychosis: A qualitative analysis

**DOI:** 10.1192/j.eurpsy.2022.536

**Published:** 2022-09-01

**Authors:** E. Csipke, F. Lammas, A. Phillips, S. Dopson, E. Joyce, T. Wykes

**Affiliations:** 1 Kings College London, Institute of Psychiatry, Psychology and Neuroscience, Psychology, London, United Kingdom; 2 University of Oxford, Said Business Schoool, Oxford, United Kingdom; 3 University College London, Institute Of Neurology, London, United Kingdom; 4 King’s College London, Institute Of Psychiatry, Psychology And Neuroscience, London, United Kingdom

**Keywords:** Psychosis, organisational climate, Cognitive remediation, early intervention

## Abstract

**Introduction:**

Cognitive remediation (CR) therapy for psychosis significantly improves recovery but is yet to be widely implemented in UK National Health Service and it is likely to be of greatest value if implemented early. Organisational climate within teams in the health services is one factor likely to affect CR implementation into Early Intervention Services (EIS), that serve those with a first episode.

**Objectives:**

To understand the organisational climate within UK NHS EIS and the barriers and facilitators for the introduction of CR.

**Methods:**

We conducted semi structured interviews with 42 EIS members of four teams in four NHS Mental Heath Trusts.

**Results:**

There were differences between teams, including involvement in decision making, leadership style, and willingness to adopt CR. Resource shortages were considered the main implementation barrier across all teams. The evidence for CR benefits and the recognition of a clinical need was the main facilitator. Teams with more democratic leadership, involving all team members in decision making, and knowledge of both the evidence base and need for CR, may feel better able to successfully incorporate it into their service.

**Conclusions:**

Engaging team members in the implementation process through cooperative and consultative decision-making can stimulate a flattened hierarchical structure, empowering staff to overcome existing and new NHS pressures and effectively deliver evidence-based care. The consideration of local conditions and organisational micro-climates mediate the successful implementation of new interventions and is needed in addition to generic, context-free variables such as resources before new interventions can be introduced.

**Disclosure:**

No significant relationships.

